# Salidroside derivative SHPL-49 enhances synaptic remodeling in BCCAO rats via the CDK5/p35/p25 signaling pathway

**DOI:** 10.3389/fphar.2026.1727177

**Published:** 2026-03-23

**Authors:** Pei Zhang, Hui Guo, Yue Shen, Ruyi Wang, Shoujiao Peng, Ruixiang Li, Jiange Zhang

**Affiliations:** The Research Center of Chiral Drugs, Innovation Research Institute of Traditional Chinese Medicine (IRI), Shanghai University of Traditional Chinese Medicine, Shanghai, China

**Keywords:** CDK5, ischemic stroke, p25, p35, SHPL-49, synaptic remodeling

## Abstract

**Background:**

In recent years, significant attention has been directed toward the development of therapeutic agents targeting cognitive and motor dysfunction following ischemic stroke. Our research team synthesized SHPL-49, a salidroside derivative, which has demonstrated neuroprotective effects in rat models of ischemic stroke in prior studies. However, the underlying mechanisms remain poorly understood. This study aims to evaluate the therapeutic potential of orally administered SHPL-49 over a 28-day period in rats with bilateral common carotid artery occlusion (BCCAO), specifically focusing on improvements in cognitive and motor deficits, and to further elucidate its mechanism of action.

**Methods:**

A BCCAO rat model was employed to investigate the effects of SHPL-49 on cognitive and motor functions following ischemic stroke. Behavioral performance was assessed using the Morris water maze, shuttle box, running wheel, gait analysis, and rotarod tests. Synaptic remodeling in the brain was evaluated through Golgi staining, Western blotting, and immunofluorescence. *In vitro* transcriptomic changes in PC-12 cells were analyzed by RNA sequencing to identify SHPL-49-regulated genes associated with synaptic remodeling. Furthermore, primary neurons exposed to oxygen-glucose deprivation/reperfusion (OGD/R) were utilized to validate the potential therapeutic targets of SHPL-49.

**Results:**

SHPL-49 significantly ameliorated cognitive and motor impairments in BCCAO rats. SHPL-49 markedly increased dendritic spine density in brain tissue and promoted synaptic remodeling by upregulating the mRNA expression of synapse-related genes, including MAP-2, SYP, and βIII-tubulin, as well as the expression of the key synaptic protein SYN1. Furthermore, transcriptomic analysis of SHPL-49-treated PC-12 cells suggested that SHPL-49 may regulate synaptic remodeling through CDK4 and CDK5 signaling. Subsequent experiments confirmed that SHPL-49 enhanced the expression of CDK5 and its activator p35 in both primary neurons and BCCAO rats, meanwhile inhibited the cleavage of p35 into p25 (a proteolytic event associated with dendritic spine atrophy and synaptic protein degradation).

**Conclusion:**

In summary, SHPL-49 significantly improved memory and motor impairments in BCCAO rats, highlighting the role of synaptic remodeling in this therapeutic effect. The underlying mechanism is closely linked to the modulation of CDK5/p35/p25 signaling pathway. These findings provide important theoretical evidence supporting the potential for the long-term clinical oral application of SHPL-49 in treating ischemic stroke.

## 1 Introduction

Ischemic stroke is a leading cause of disability and mortality worldwide (Herpich and Rincon, 2020; Zhao et al., 2022). Its high incidence and substantial disability rate impose a significant burden on both society and families (Kolmos et al., 2021; Tu and Wang, 2023). The onset of brain ischemia can trigger a cascade of pathological and physiological alterations, including energy metabolism disorders, oxidative stress, inflammatory responses, and apoptosis. The main manifestation including cerebral hypoperfusion and neuronal death, ultimately resulting in cognitive and motor impairments that behaviorally compromise the quality life of patients (Kim et al., 2025). As critical structures for neuronal signal transmission, synapses are essential for maintaining neural circuit function. Research has demonstrated that ischemic injury induces significant synaptic damage, disrupting the release, synthesis, and transport of neurotransmitters. These disturbances further exacerbate neuronal injury, contributing to deficits in cognition, memory, and motor function (van Oostrum and Schuman, 2025). Following treatment, surviving neurons may form new synaptic connections, which undergo structural modifications through synapses remodeling, thereby promoting neural development and functional recovery of cognition and motor (Lee et al., 2024). Thereby, synaptic remodeling represents a key adaptive mechanism of the central nervous system, which has emerged as a promising therapeutic strategy for improving neurological outcomes in ischemic stroke.

Currently, the clinical management of neurological function recovery following ischemic stroke primarily involves the integration of pharmacological interventions and rehabilitation training (Ho and Powers, 2025; Paul and Candelario-Jalil, 2021). For example, the free radical scavenger edaravone facilitates synaptic remodeling by reducing oxidative stress, whereas butylphthalide promotes neuronal survival and synaptic reconstruction by upregulating neurotrophic factor expression (Li et al., 2023; Xu et al., 2022). In addition, rehabilitation strategies such as motor therapy and cognitive training contribute to improved cerebral perfusion and enhanced synaptic remodeling, thereby supporting functional recovery (Hortobágyi et al., 2022; Kwakkel et al., 2023). However, most existing drugs act through relatively narrow mechanisms, making it challenging to comprehensively modulate the complex molecular pathways and microenvironmental networks involved in synaptic remodeling, thus limiting their therapeutic efficacy (Sun et al., 2024; Zhang et al., 2025). Furthermore, certain medications are associated with adverse effects, including hepatic and renal dysfunction as well as gastrointestinal discomfort (Settanni et al., 2021). Therefore, the development of novel therapeutic agents with greater safety and broader regulatory capacities is of considerable clinical significance.

Salidroside, the primary active constituent extracted from *Rhodiola rosea* L., has shown significant pharmacological activities, including antioxidant, anti-aging, and anti-inflammatory properties. SHPL-49 is a novel compound synthesized by our research group through structural modification of salidroside (Zhang et al., 2023). Previous studies have demonstrated that SHPL-49 significantly reduces the volume of cerebral infarction in rats subjected to permanent middle cerebral artery occlusion (pMCAO) model on days 5 and 14, and promotes neuronal survival (Zhao et al., 2024). In preliminary study on neuroprotective mechanism, SHPL-49 can enhance neuronal survival through attenuating glutamate excitotoxicity, alleviating calcium overload and oxidative stress, and inhibiting cell apoptosis (You et al., 2024). In addition, SHPL-49 enhanced synaptic plasticity in primary neurons by promoting growth-associated protein 43 (Gap43) expression via brain-derived neurotrophic factor (BDNF) signaling pathway (You et al., 2024). However, in the aforementioned study, we only investigated the neuroprotective effects of SHPL-49 in pMCAO rats following administration via tail vein injection or oral gavage over a 14-day period; its reparative effects and underlying mechanism in long-term ischemic brain injury remain unclear. Therefore, in this study, we continue to employ oral administration to evaluate the cognitive and motor functional improvements induced by SHPL-49 at a dose of 90 mg/kg over 28 days in rats subjected to bilateral common carotid artery occlusion (BCCAO), and further explored the underlying mechanisms of action, with salidroside used as a positive control. Additionally, a primary neuronal injury model was established using oxygen-glucose deprivation/reoxygenation (OGD/R) to further assess the neuroprotective effects of SHPL-49. The aim was to elucidate the mechanism underlying SHPL-49-mediated synaptic remodeling, neurofunctional recovery, and modulation of associated signaling pathways, thereby providing a robust theoretical foundation for its clinical translation research.

## 2 Materials and methods

### 2.1 Animals

Male Wistar rats (230–270 g) were purchased from Beijing Vital River Laboratory Animal Technology Co., Ltd. (Beijing, China). Sprague-Dawley (SD) female rats at 16–18 days of gestation were obtained from Shanghai Jihui Laboratory Animal Care Co., Ltd. (Shanghai, China). All animal experimental protocols were approved by the Animal Care and Use Committee at Shanghai University of Traditional Chinese Medicine, China (Ethics number: PZSHUTCM2302020001, PZSHUTCM2303070001). The experiments were performed in strict accordance with the Guide for the Care and Use of Laboratory Animals published by the National Institutes of Health and the ARRIVE guidelines. Rats were housed in a climate-controlled room (temperature: 22 °C–26 °C; relative humidity: 40%–70%) under a 12 h light/dark cycle and had free access to standard chow and water.

### 2.2 Animal model of bilateral common carotid artery occlusion (BCCAO)

The BCCAO model was established in accordance with previously published protocols (Xiao et al., 2022). Briefly, The rats were anesthetized with 3% isoflurane, followed by maintenance with 2.5% isoflurane. An midline cervical incision approximately 1 cm length was made, and the subcutaneous tissue was bluntly dissected. The bilateral common carotid arteries and vagus nerves were carefully isolated, and the arteries were ligated with 4–0 silk sutures. In the sham-operated group, the same surgical procedure was performed without ligation of the carotid arteries. Throughout the surgical procedure, rectal temperature was maintained at 36.5 °C–37.5 °C using a heating pad. The incisions were sutured, and cerebral blood flow was monitored using a laser Doppler flowmeter (Moor VSM, United Kingdom). The BCCAO model was considered successfully established when cerebral blood flow in the model group dropped to 50% of baseline levels (Farkas et al., 2007).

### 2.3 Experimental groups

The randomization tool in GraphPad (San Diego, CA) was employed to assign rats to different experimental groups, and coding was implemented during surgery to ensure blinding during data analysis. All rats received treatment 30 minutes after occlusion. SHPL-49 (Batch Number: 3001430101) was provided by Shanghai Hutchison Pharmaceuticals Limited. Salidroside (SAL Batch Number: 202210) was supplied by Shanghai XianDinn Biotech Co. Ltd. SAL In this study, the dosage of SHPL-49 (90 mg/kg) was determined based on findings from our previous study (Wang et al., 2025b). In the experiment, 48 rats were treated for 28 days following model induction, as follows: (1) Sham group, n = 12; (2) Model group, n = 12; (3) SHPL-49 group (90 mg/kg), n = 12; (4) SAL Group (90 mg/kg), n = 12. Following drug administration, six animals were randomly selected from each group (n = 12 per group) for behavioral assessments. The Morris water maze test was conducted from days 24–28 post-administration. Shuttle box and rotarod tests were performed on days 7, 14, 21, and 28. Running wheel activity was evaluated from days 25–28, and gait analysis was carried out on day 28. Upon completion of the behavioral testing period, these animals were transcardially perfused and subsequently euthanized. Their brains were promptly harvested, sectioned, and processed for histopathological examination and immunofluorescence staining. The remaining six animals in each group were euthanized on day 28 post-administration, and brain tissues were collected for subsequent molecular analyses, including qRT-PCR and Western blotting. Each group received oral administration at the same time each day for 28 consecutive days. The sham-operated group and the model groups were administered normal saline in parallel with the treatment groups. Behavioral assessments were conducted during daylight hours.

### 2.4 Morris water maze (MWM) experiment

The Morris water maze test was conducted over five consecutive days, starting on day 24. Each rat was placed into the maze from one of four designated entry points on the pool wall each day. The swimming trajectory, escape latency, and total swimming distance within 90 s were recorded to evaluate the rats’ learning ability in spatial orientation. On day 28, a spatial exploration test was performed, during which the hidden platform was removed. Each rat was introduced into the water from the entry point farthest from the original platform location, and the time spent in the target quadrant, the number of platform crossings, and the swimming path within 90 s were recorded to assess memory retention.

### 2.5 Shuttle box experiment

A two-way shuttle box (SANS, China) was employed to evaluate the learning and memory performance of rats. Prior to the experiment, the rats were allowed a 10-min adaptation period to acclimate to the shuttle box environment. Active avoidance behavior was defined as a rat successfully transferring to the safe compartment in response to auditory and visual stimuli before the delivery of an electric foot shock. Passive avoidance behavior was recorded when a rat entered the safe compartment during the shock stimulus. Failure to reach the safe compartment before the shock terminated was classified as an error. A lower error count reflected better learning and memory capacity. The effects of SHPL-49 on cognitive function were assessed by recording behavioral data on the days 7, 14, 21, and 28 after treatment initiation.

### 2.6 Wheel running experiment

The running wheel system (SANS, China) was used to assess the recovery of motor function in BCCAO rats. The rats were placed in the running wheel apparatus and provided with *ad libitum* access to food and water. The total distance traveled, duration of movement, and average speed over a 1-h period were recorded.

### 2.7 Gait experiment

The rats underwent a 3-day adaptive training period to ensure stable and continuous locomotion along the runway. At the start of each trial, the animals positioned at the beginning of the runway, and video recording commenced upon their release. Each rat was allowed 10 s to traverse the runway during a trial, and each experiment was conducted in triplicate. Following data acquisition, the VisuGait system (Xinruan Company, China) was employed to identify and analyze the effective frames for gait assessment. Finally, gait trajectory diagrams, step sequence diagrams, gait imbalance coefficients, and the proportion of normal step sequences were subsequently subjected to statistical analysis.

### 2.8 Rota-rod test

Subsequently, these rats underwent a 3-day adaptive training period. During the training period, the speed of the rotating rod was set at 8 rpm, and the rats were allowed to train on the apparatus for 5 min each day. Rota-rod tests (Panlab, Spain) were conducted on days 7, 14, 21, and 28. At the beginning of the formal experiment, the rotating rod apparatus accelerated uniformly from an initial speed of 4 rpm to a maximum of 40 rpm. The duration each rat stayed on the rotating rod from the moment of placement until falling off (referred to as the stay time), as well as the rotational speed at the moment of falling (fall speed), were recorded. If a rat remained on the rotating rod for 5 min without falling, the stay time was recorded as 5 min and the fall speed as 40 rpm. All data were collected using a double blind procedure objectivity and reliability of the experimental outcomes.

### 2.9 H&E staining and Nissl staining

The rats were anesthetized with isoflurane and subsequently subjected to transcardial perfusion with 0.9% normal saline and 4% paraformaldehyde. The brains were carefully excised and post-fixed in 4% paraformaldehyde for 48 h. All brain tissues were then embedded in paraffin and sectioned into 5-μm-thick coronal slices. The paraffin sections of brain tissue were stained with hematoxylin and eosin (H&E) and Nissl stain to evaluate histological features and the distribution of Nissl bodies in the hippocampal CA3 region under an optical microscope (Olympus Corporation, Japan).

### 2.10 Golgi-Cox staining

Whole rat brain tissues were promptly harvested and immersed in the designated Golgi-Cox fixative. Staining procedures were carried out according to the instructions provided by the manufacturers of the FD Rapid Golgi-Cox Staining Kit (FD Neuro Technologies, PK401, United States). Pyramidal neurons located in the hippocampal region were examined, and three basal dendritic segments per neuron, each with a minimum length of 30 μm, were randomly selected for analysis. Dendritic spine density was quantified using ImageJ software (Version 1.8.0).

### 2.11 RNA sequencing and bioinformatics analysis

PC-12 cells (a highly differentiated cell line derived from the rat adrenal medulla and originally isolated from pheochromocytoma tissue, widely used as a neuronal model in neuroscience research) were purchased from Shanghai Zhongqiao Xinzhou Biotechnology Co., Ltd. The cells were cultured in DMEM medium (Gibco, United States) supplemented with 10% fetal bovine serum (FBS) (SERANA, Germany) with 5% CO_2_ at 37 °C. For experimental procedures, PC-12 cells were seeded into 6-well plates at a density of 2.5 × 105 cells per well in 2 mL of culture medium and incubated overnight to allow for complete cell adhesion. Subsequently, the supernatant was removed from each well. RNA samples were then prepared and divided into two groups: (1) control group (n = 4); (2) SHPL-49 group (150 μM, n = 5). RNA sequencing and bioinformatics analysis were performed by a commercial service provider (Novogene Co. Ltd., China). Briefly, total RNA was extracted, and the transcriptome sequencing was performed based on the Illumina sequencing platform. Significant differences in gene expression were identified using the EdgeR algorithm implemented in the CLC Genomics Workbench (Qiagen, Hilden, Germany). The *P* value threshold was adjusted for multiple testing using the false discovery rate (FDR) method. An FDR threshold of <0.05 was adopted to judge the statistical significance of gene expression change across the entire tripartite bioassay.

### 2.12 Oxygen-Glucose Deprivation and Reoxygenation (OGD/R) model

Primary cortical neurons were isolated from E16-E18 rat embryos using a protocol identical to that previously described (Xie et al., 2024). The isolated cortical tissues were digested for 30 min at 37 °C in cell culture medium supplemented with papain (2 mg/mL, Sangon, China) and DNase I (0.5 mg/mL, Meilunbio, China). The resulting cell suspension was diluted with neurobasal medium supplemented with B27 (Gibco, United States), and then seeded into poly-L-lysine-coated 6-well plates (Beyotime Biotechnology, China) at a density of 2.5 × 10^^5^ cells per well. Oxygen-Glucose Deprivation and Reoxygenation (OGD/R) was used to simulate cerebral ischemia-reperfusion in the cell model. After washing cells with phosphate-buffered saline (PBS) (HAKATA), the media was replaced with sugar-free and serum-free 1,640 medium in the model group (HAKATA). For the drug administration groups, the media was replaced with sugar-free serum free medium containing the drug. Hypoxia was achieved using a hypoxic tank connected to a hypoxic apparatus, gas was replaced with 95% N2, 4% CO_2_, and 1% O_2_. After gas replacement, the hypoxic tank was placed in the cell culture chamber (Thermo). Primary neurons were cultured under hypoxic conditions for 4 h. After the culture is completed, remove the culture vessels from the hypoxic chamber and quickly transfer them to an incubator set at 37 °C and 5% CO_2_. In the conventional incubator, each well was replaced with 2 mL of complete neuronal culture medium containing appropriate nutrients for reoxygenation and reglucose treatment for 24 h. For the drug administration group, the culture medium was replaced with complete neuronal culture medium containing the drug, and reoxygenation and re-glucose treatment were carried out for 24 h. To elucidate the molecular mechanism underlying SHPL-49 - mediated neuroprotection, two complementary experimental series were conducted in primary cortical neurons subjected to OGD/R. In the first series, we evaluated the impact of SHPL-49 on calpain - dependent p35 cleavage. Neurons were randomized into six treatment groups: (1) untreated control; (2) OGD/R only (injury model); (3) SHPL-49 at 50 μM; (4) SHPL-49 at 100 μM; (5) SHPL-49 at 150 μM; and (6) PD151746 (20 μM), a specific calpain inhibitor that prevents the cleavage of p35 into p25 and thus served as a mechanistic positive control. In the second series, we tested whether the effects of SHPL-49 are dependent on CDK5 kinase activity. Neurons were assigned to four groups: (1) untreated control; (2) OGD/R only (model); (3) SHPL-49 (150 μM); and (4) SHPL-49 (150 μM) co-administered with Roscovitine (20 μM), a well characterized ATP-competitive CDK5 inhibitor (Gao et al., 2020). Following treatment, cells were harvested for quantitative assessment of synaptic protein expression by Western blotting and mRNA levels by quantitative reverse transcription polymerase chain reaction (qRT-PCR).

### 2.13 Real-time quantitative PCR (qRT-PCR)

The mRNA was extracted from primary neurons, and its concentration was quantified using a spectrophotometer (Thermo Fisher Scientific, United States). Complementary DNA (cDNA) was synthesized through reverse transcription, and quantitative real-time PCR (qRT-PCR) was subsequently performed on an ABI QuantStudio™ 6 System (Thermo Fisher Scientific, United States) using ChamQ Universal SYBR qPCR Master Mix (Vazyme, Nanjing, China). β-actin was used as the internal reference gene, and relative gene expression levels were calculated using the 2^−ΔΔct^ method. The primer sequences are listed in Table 1.

**TABLE 1:** Sequences of primers used for real-time quantitative polymerase chain reaction.

Gene	Sequences (5′→ 3′)
β-actin	F-AGAGGGAAATCGTGCGTGAC
	R-CCATACCCAGGAAGGAAGGCT
βIII-tubulin	F-GCCATACGCATCTACGACCT
	R-TCCAGGAAGGACACCTCGTC
MAP-2	F-AGTGGTGAATCAGCTCAGGC
	R-GGGAGGATGGAGGAAGGTCT
SYP	F-TCTTCGCCTTTGCTACGTGT
	R-AGATCTTGGTAGTGCCCCCT
CDK4	F-TGCAACGCCTGTGGATATGT
	R-AGGCTCCTCGAGGAAGAGAG
CDKn2c	F-TCATTCACGATGCTGCCAGA
	R-CATTGCAGGCTGTGTGCTTC
CDK5	F-GTATCCCAGTCCGCTGCTAC
	R-ACATCATTGCCGGGGAAGAG
CDK5r1	F-CCAGCTATCGAAAGGCCACA
	R-CCTCTTCCAAGGCAGTACCG

### 2.14 Western blot

Protein samples were extracted from brain tissue and primary neurons, and their concentrations were determined using a BCA protein assay kit (Meilunbio, China). Equal amounts of protein were separated by SDS-PAGE and transferred onto polyvinylidene diffuoride (PVDF) membranes (Bio-Rad, United States). Membranes were blocked with 5% non-fat milk (Beyotime, China) in Tris-buffered saline containing 0.1% Tween-20 (TBST) and incubated overnight at 4 °C with the following primary antibodies: SYP (1:1,000, CST, United States), SYN1 (1:1,000, Boster, China), PSD95 (1:1,000, ABCAM, United States), CDK5 (1:1,000, Boster, China), p35/25 (1:1,000, CST, United States), p-PSD95 (1:1,000, CST, United States). Following washing, membranes were incubated for 2 h at room temperature with HRP-conjugated goat anti-rabbit IgG (H + L) (1:5,000, Beyotime, China) or mouse anti-mouse IgG (H + L) (1:5,000, Beyotime, China) as secondary antibodies. Immunoreactive bands were visualized using enhanced chemiluminescence (ECL) detection reagents (Meilunbio, China) and semi-quantitatively analyzed by ImageJ software (Chatterjee et al., 2022).

### 2.15 Immunofluorescent staining

Brain tissue sections were immunostained as previously described. Paraffin sections were deparaffinized, rehydrated through a graded ethanol series following xylene treatment, and blocked with 10% goat serum for 1 h at room temperature. The sections were incubated overnight at 4 °C with the following primary antibodies: NeuN (1:500, Oasis, China), SYP (1:200, CST, United States), SYN1 (1:200, Boster, China), PSD95 (1:200, ABCAM, United States), GFAP (1:200, Abcam, United States) and CSPG (1: 200, Sigma, United States). After washing with PBS, sections were incubated for 2 h at room temperature with secondary antibodies: goat anti-mouse IgG H&L (Alexa Fluor® 488) (1:500, Abcam) or donkey anti-rabbit IgG H&L (Alexa Fluor® 555) (1:500, Abcam). Following three washes with PBS (Servicebio), sections were were mounted with an anti-fade mounting medium (Beyotime) containing 4, 6-diamidino-2-phenylindole (DAPI) and coverslipped. Immunofluorescence images were acquired using a laser scanning confocal microscope (Nikon).

### 2.16 Determination of calpain activities

Brain tissues and primary cortical neurons were homogenized on ice in cold lysis buffer. Calpain activity was quantified in tissue and cell lysates using a commercially available fluorometric assay kit (P0375S, Beyotime). Briefly, lysates were centrifuged at 12,000 × g for 15 min at 4 °C to obtain clear supernatants. Protein concentration was determined using the BCA assay, and lysate concentrations were normalized to 1 mg/mL with lysis buffer. Subsequently, 5 μL of the calpain-specific fluorogenic substrate suc-LLVY-AMC was added to each well. Samples were incubated at 37 °C for 1 h in the dark, and fluorescence intensity was measured using a microplate reader. Calpain activity was calculated as relative fluorescence units (RFU) per μg protein per hour, with background fluorescence from blank wells subtracted.

### 2.17 Statistical analysis

All data are presented as means ± standard deviations (mean ± SD). Unpaired two-tailed t-tests were performed for comparisons between two groups, and multiple comparisons were conducted using one-way analysis or two-way analysis of variance (ANOVA) using GraphPad Prism 8 (GraphPad Software, California, United States). For behavioral assays involving repeated measurements across multiple time points - including shuttle tests and rod-turning tests (assessed on days 7, 14, 21, and 28) and the voluntary running wheel tests (recorded daily from day 25 to day 28) - a two-way repeated measures analysis of variance was employed. Time was designated as the within-subjects factor, and the various treatment group was treated as the between-subjects factor. When a statistically significant interaction between time and treatment was observed, post hoc pairwise comparisons were performed with appropriate correction for multiple testing. *P* values <0.05 was considered statistically significant.

## 3 Results

### 3.1 SHPL-49 enhances the recovery of cognitive function in BCCAO rats

**FIGURE 1:**
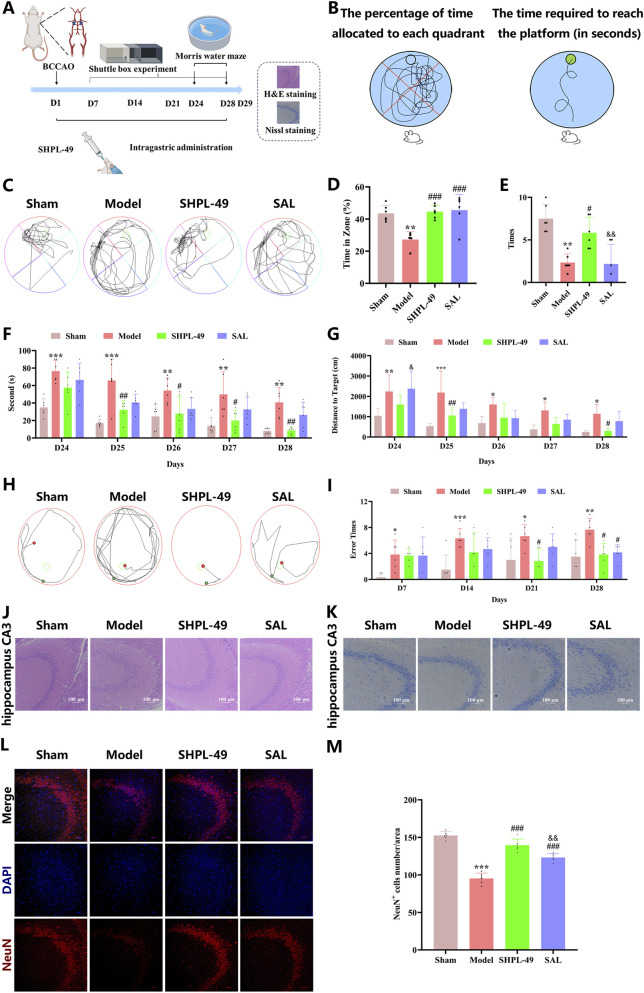
The cognitive and mnemonic effects of SHPL-49 were evaluated in a rat model of BCCAO following 28 days of continuous administration. **(A)** Flowchart of the experimental design. **(B)** Schematic illustration of the Morris Water Maze (MWM) setup. **(C)** Trajectory plots representing the spatial navigation phase recorded during the MWM task. **(D)** Quantitative analysis of the percentage of time spent in the target quadrant during the spatial exploration test in the MWM. **(E)** Quantification of platform crossings during the spatial probe test in the MWM. **(F)** Quantitative analysis of escape latency during the positioning navigation phase of the MWM. **(G)** Quantitative analysis of total swimming distance during the positioning navigation phase of the MWM. **(H)** Trajectory tracks recorded during the localization navigation phase of the MWM. **(I)** Quantification of error frequency in the shuttle box avoidance test. **(J)** Representative hematoxylin-eosin (H&E) staining images of the hippocampal CA3 region in the brain tissue of BCCAO rats 28 days post-modeling. **(K)** Representative Nissl staining images of the hippocampal CA3 region in the brain tissue of BCCAO rats 28 days post-modeling. **(L)** Representative immunofluorescence images showing neuronal nuclear antigen NeuN (red) in the hippocampal CA3 region of brain tissue from BCCAO rats at 28 days post-modeling. Nuclei were counterstained with DAPI (blue). Scale bars = 50 μm. **(M)** Quantitative analysis of NeuN-positive neurons based on immunofluorescence staining. Data are expressed as mean ± SD. *P < 0.05, **P < 0.01, ***P < 0.001, Model group vs. Sham group; ^#^ *P* < 0.05, ^##^ *P* < 0.01, ^###^ *P* < 0.001, SHPL-49 group or SAL group vs. Model group; ^&&^ *P* < 0.01, SHPL-49 group vs. SAL group. For the Morris water maze, shuttle box task, and H&E, Nissl and NeuN staining, n = 6 per group.

To evaluate the effect of SHPL-49 on cognitive function recovery following ischemic stroke, BCCAO rats were orally administered SHPL-49 (90 mg/kg), SAL (90 mg/kg), or normal saline. Morris Water Maze (MWM) testing was conducted from day 24 to day 28, and shuttle box experiments were carried out on days 7, 14, 21, and 28 (Figures 1A,B). In the MWM spatial exploration test, SHPL-49 treatment significantly increased the time spent in the target quadrant after 28 days of administration (*P* < 0.001), with an efficacy comparable to that of SAL SHPL-49 also significantly increased the number of platform crossings. However, no significant difference in platform crossings was observed between the SAL group and the Model group (Figures 1C–E). In the place navigation test, SHPL-49 significantly reduced escape latency from day 25 to day 28 and shortened swimming distance on days 25 and 28 (*P* < 0.05, *P* < 0.01), whereas no significant differences were found between the SAL group and the Model group (Figures 1F–H). In the shuttle box experiment, the number of errors in the SHPL-49 group was significantly reduced on day 21 and day 28 (*P* < 0.05). On day 21, no significant difference was observed between the SAL group and the model group; however, a significant difference emerged between these two groups on day 28 (*P* < 0.05). The performance of the SHPL-49 group was comparable to that of the normal group on days 21 and 28. In contrast, no significant differences were detected between the drug-treated groups and the model group on days 7 and 14 (Figure 1I). H&E staining revealed that neurons in the CA3 regions of the hippocampus in the SHPL-49 group were more densely packed and orderly arranged compared to those in the model group, with an effect similar to that observed in the SAL group (Figure 1J). Nissl staining revealed that, in the SHPL-49 group, the number of shrunken and degenerated neurons in the hippocampal CA3 region was markedly reduced, with well-preserved Nissl bodies, demonstrating a neuroprotective effect comparable to that observed in the normal group (Figure 1K), indicating that SHPL-49 effectively promoted the recovery of the neuronal protein synthesis machinery. In addition, the results of NeuN immunofluorescence staining demonstrated a significant increase in the number of neurons in the CA3 region of the hippocampus in the SHPL-49 group (*P* < 0.001) (Figures 1L,M), indicating that SHPL-49 exerts a robust neuroprotective effect in the chronic cerebral ischemia model and can effectively mitigate neuronal loss.

### 3.2 SHPL-49 promotes the recovery of motor coordination of BCCAO rats

**FIGURE 2:**
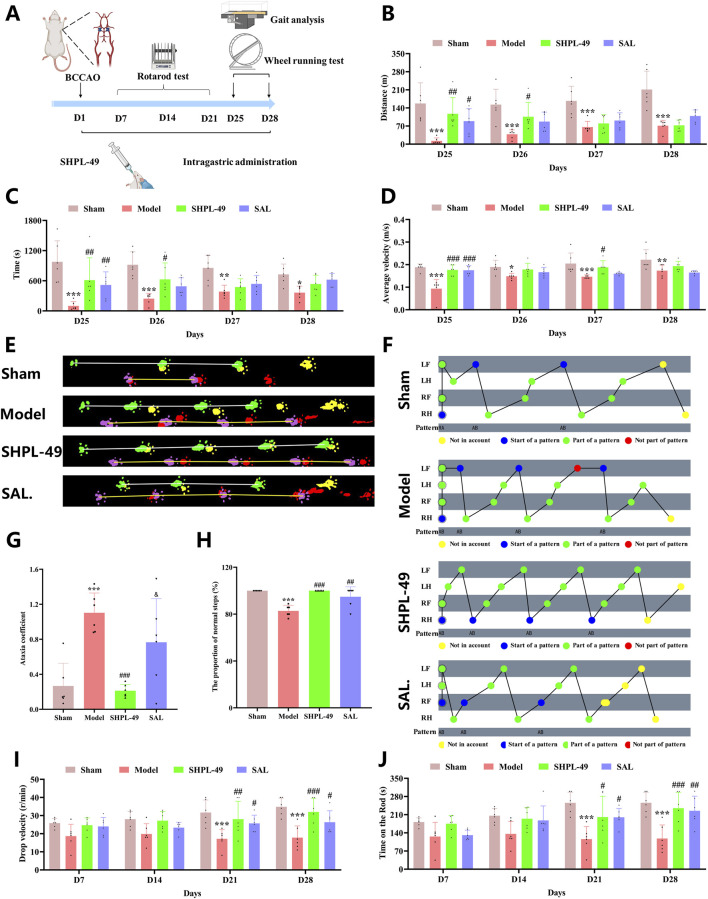
Evaluation of motor coordination ability in BCCAO rats after 28 days of continuous SHPL-49 administration. **(A)** Flowchart of the animal experiment. **(B)** Quantification of running distance in the running-wheel test. **(C)** Quantification of running duration in the running-wheel test. **(D)** Quantification of average speed in the running wheel test. **(E)** Movement trajectory chart from the gait analysis test. **(F)** Footprint sequence diagram from the gait analysis test. **(G)** Quantification of the gait incoordination coefficient. **(H)** Quantification of the proportion of normal gait sequences. **(I)** Quantification of fall latency in the rotarod fatigue test. **(J)** Quantification of retention time on the rotarod in the rotarod fatigue test. Data are expressed as mean ± SD. *P < 0.05, **P < 0.01, ***P < 0.001, Model group vs. the Sham group; ^#^ *P* < 0.05, ^##^ *P* < 0.01, ^###^ *P* < 0.001, SHPL-49 group or SAL group vs. the Model group. n = 6 per group; ^&^ *P* < 0.05, SHPL-49 group vs. SAL group.

To evaluate the effect of SHPL-49 on motor function after ischemic stroke, a series of behavioral tests were conducted. The running wheel test was performed from day 25 to day 28, the gait test was conducted on day 28, and the rotarod test was carried out on days 7, 14, 21, and 28 (Figure 2A). In the running wheel experiment, the running distance, duration and average speed in the SHPL-49 group on the day 25 were significantly higher than those of the model group and comparable to those of the SAL group. On the day 26, the running distance and duration in the SHPL-49 group were significantly greater than those of the model group. On the day 27, the average speed in the SHPL-49 group was significantly higher than that of the model group, whereas the SAL group showed no significant difference compared to the model group from day 26 to day 28 (*P* < 0.05, *P* < 0.01, *P* < 0.001) (Figures 2B–D). To further evaluate its impact on motor coordination, gait analysis was performed in rats. The results demonstrated that SHPL-49 significantly improved locomotor activity and gait sequence pattern regularity (Figures 2E,F). The gait disorder coefficient was markedly reduced in the SHPL-49 group (*P* < 0.001), with no significant difference observed between the SAL group and the model group (Figure 2G). Additionally, the proportion of normal gait patterns increased in the SHPL-49 group (*P* < 0.01, *P* < 0.001), reaching levels comparable to those in the SAL group (Figure 2H). In the rotarod test, the latency to fall in the SHPL-49 group was significantly prolonged on day 21 and 28, and the rotational speed at the time of fall was increased, comparable to that in the SAL group (*P* < 0.05, *P* < 0.01, *P* < 0.001) (Figures 2I,J). In conclusion, these findings confirm that SHPL-49 promotes the recovery of motor function in rats following ischemic stroke.

### 3.3 SHPL-49 promotes the plasticity of synapse

**FIGURE 3:**
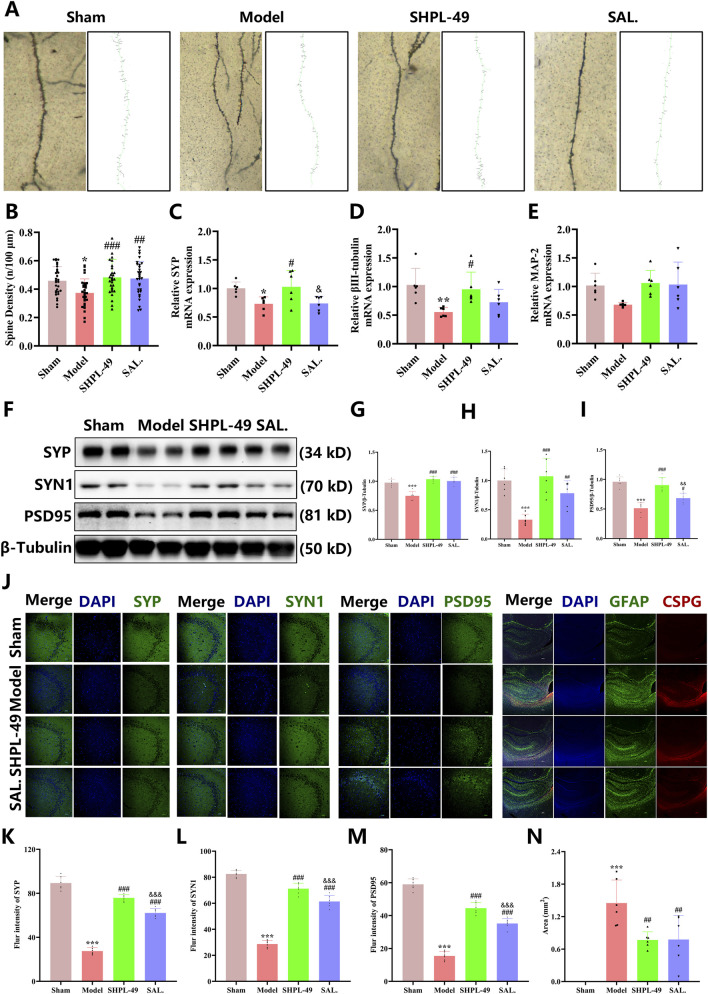
SHPL-49 at a dosage of 90 mg/kg enhances synaptic plasticity. **(A)** Representative Golgi-Cox staining images of rat brain tissue. **(B)** Quantitative analysis of dendritic spine density. **(C–E)** qRT-PCR analysis of key markers associated with synaptic remodeling: SYP, β-III tubulin and MAP-2. **(F)** Representative Western blot images showing the expression of presynaptic markers SYP and SYN1, as well as the postsynaptic marker PSD95, in rat brain tissue. **(G)** Quantification of SYP protein levels in rat brain tissue. **(H)** Quantification of SYN1 protein levels in rat brain tissue. **(I)** Quantification of PSD95 protein levels in rat brain tissue. **(J)** Immunofluorescence staining image of SYP, SYN1, PSD95, and the glial scar. Scale bars = 50 μm for synaptic markers; and 200 μm for the glial scar. **(K)** Quantification of SYP fluorescence intensity based on immunofluorescence staining. **(L)** Quantification of SYN1 fluorescence intensity based on immunofluorescence staining. **(M)** Quantification of PSD95 fluorescence intensity based on immunofluorescence staining. **(N)** Quantification of glial scar area based on immunofluorescence Staining. Data are presented as mean ± SD. *P < 0.05, **P < 0.01, ***P < 0.001, Model group vs. the Sham group, ^#^ *P* < 0.05, ^##^ *P* < 0.01, ^###^ *P* < 0.001, SHPL-49 group or SAL group vs. the Model group. n = 6 per group; ^&^ *P* < 0.05, ^&&&^ *P* < 0.001, SHPL-49 group vs. SAL group.

To investigate whether SHPL-49 mediates neuroprotective effects via synaptic remodeling, we conducted Golgi-Cox staining. The results demonstrated that 28 days after BCCAO, SHPL-49 significantly enhanced dendritic spine density (*P* < 0.01, *P* < 0.001) (Figures 3A,B). Subsequently, we assessed the mRNA expression levels of synapse-associated genes, including SYP, βIII-tubulin and MAP-2, in rat brain tissues. These gene transcripts were markedly downregulated in the BCCAO group compared to the control group (*P* < 0.05, *P* < 0.01). Notably, SHPL-49 treatment reversed this decline, with SYN1 and βIII-tubulin mRNA levels in the SHPL-49 group surpassing those in the positive control group treated with SAL (*P* < 0.05) (Figures 3C–E). These findings suggest that SHPL-49 promotes synaptic remodeling. To further validate this effect, we performed Western blot analysis to assess the expression of SYN1. The results demonstrated a significant increase in the presynaptic markers SYP and SYN1, as well as the postsynaptic marker PSD95, at the protein level following SHPL-49 treatment (*P* < 0.05, *P* < 0.01, *P* < 0.001) (Figures 3F–I). Subsequently, immunofluorescence staining of SYP, SYN1 and PSD95 was examined in the CA3 region of the hippocampus. The findings revealed that SHPL-49 significantly enhanced the fluorescence signal intensity of SYP, SYN1 and PSD95 in this region (*P* < 0.01, *P* < 0.001). The glial scar represents a major barrier to neuronal synaptic remodeling. Subsequently, immunofluorescence staining was performed to assess co-localization of GFAP and CSPG. Minimal glial scarring was observed in the Sham group, whereas the Model group exhibited a markedly expanded distribution of glial scars (*P* < 0.001), indicating extensive glial scar formation in BCCAO model rats. Notably, the SHPL-49 group showed a significant reduction in glial scar area (*P* < 0.01), comparable to the effect observed in the SAL group (Figures 3J–N). Collectively, these findings suggest that SHPL-49 significantly promotes synaptic remodeling after stroke and thereby exerts neuroprotective effects.

### 3.4 SHPL-49 promotes plasticity synapse by enhancing the expression of CDK5

**FIGURE 4:**
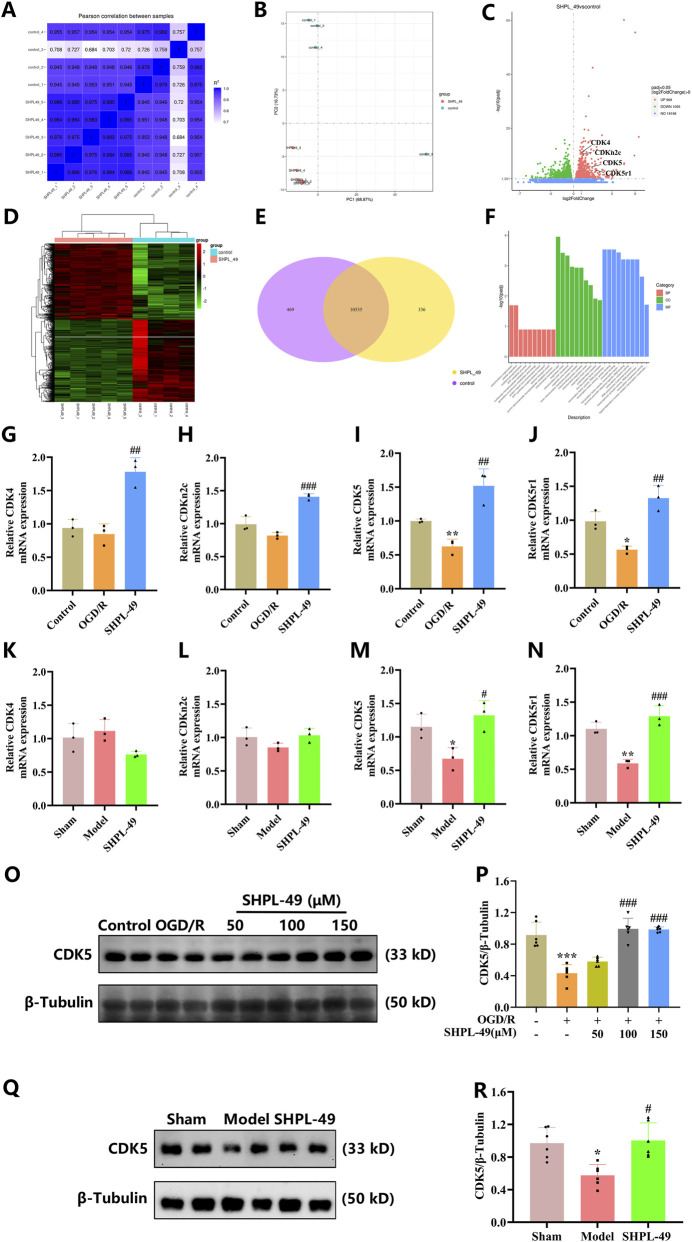
SHPL-49 promotes the expression of CDK5 at both mRNA and protein levels. **(A,B)** Correlation analysis among samples. **(C,D)** Differential expression analysis between experimental groups. **(E)** Venn diagram showing the overlap of differentially expressed genes. **(F)** GO enrichment analysis of differentially expressed genes. **(G–J)** qRT-PCR validation of selected differentially expressed genes in primary neurons. **(K–N)** qRT-PCR validation of differentially expressed genes in rat brain tissue. **(O)** Representative Western blot images of CDK5 expression in primary neurons. **(P)** Quantitative analysis of CDK5 protein levels in primary neurons. **(Q)** Representative Western blot images of CDK5 expression in rat brain tissue. **(R)** Quantitative analysis of CDK5 protein levels in rat brain tissue. Data are presented as mean ± SD. *P < 0.05, **P < 0.01, ***P < 0.001, OGD/R group vs. Control group, Model group vs. Sham group compared with the Control group, ^#^ *P* < 0.05, ^##^ *P* < 0.01, ^###^ *P* < 0.001, SHPL-49 group vs. OGD/R group. n = 3 for mRNA analysis in primary neurons and rat brain tissue. n = 6 for protein analysis in primary neurons and rat brain tissue.

To further elucidate the potential mechanism underlying SHPL-49-mediated synaptic remodeling, we conducted transcriptome analysis on PC-12 cells treated with SHPL-49. The results showing differentially expressed genes across various sample groups are presented in the figure (Figures 4A,B). The volcano plot of differentially expressed genes revealed that 969 genes were upregulated and 1,055 were downregulated in the SHPL-49 group compared to the control group (Figure 4C). A heatmap was generated to visualize the relative expression levels of these genes, highlighting their distinct expression patterns across samples (Figure 4D). Furthermore, a Venn diagram was used to visualize the overlap of differentially expressed genes among the groups (Figure 4E). Subsequently, Gene Ontology (GO) functional enrichment analysis revealed that the differentially expressed genes were significantly enriched in all three GO domains: biological process (BP), cellular component (CC), and molecular function (MF) (Figure 4F). KEGG pathway enrichment analysis revealed significant enrichment of differentially expressed genes in neuro-signaling pathways, notably the dopaminergic synapse pathway (Supplementary Figure S2A). In parallel, protein - protein interaction (PPI) network analysis was performed using the STRING database (v11.5) (Supplementary Figure S2B) with default confidence parameters. Cdk5r1 emerged as a topological hub node - evidenced by high degree, betweenness, and closeness centrality - indicating its potential regulatory centrality within the dysregulated network. Based on this screening and a comprehensive literature review, CDK4/CDKN2C and CDK5/CDK5r1 were identified as candidate gene pairs potentially involved in neuronal synaptic remodeling.

To validate their involvement, qRT-PCR was performed to measure the mRNA expression levels of these genes in primary neurons and rat brain tissues. The results showed that, in primary neurons following OGD/R, SHPL-49 significantly increased the mRNA expression levels of CDK4/CDKN2C and CDK5/CDK5r1 (*P* < 0.01, *P* < 0.001) (Figures 4G–J). In rat brain tissues, SHPL-49 treatment significantly increased the mRNA expression of CDK5 (*P* < 0.05, *P* < 0.001), whereas the mRNA expression levels of CDK4 and CDK5r1 remained unchanged (Figures 4K–N). To further investigate the effect of SHPL-49 on CDK5 expression, we examined CDK5 protein levels in both primary neurons and rat brain tissues. In primary neurons subjected to OGD/R conditions, SHPL-49 treatment (100 μM, and 150 μM) significantly increased CDK5 protein expression (*P* < 0.001) (Figures 4O,P). Subsequently, CDK5 protein expression was assessed in rat brain tissue following SHPL-49 administration. In the brain tissue of BCCAO rats, SHPL-49 treatment led to a significant increase in CDK5 protein levels (*P* < 0.05) (Figures 4Q,R). CDK5 is a crucial member of the cyclin-dependent kinase family and plays an essential role in the development and functional maintenance of the nervous system. CDK5r1 encodes p35, a specific regulatory activator of CDK5. p35 can be proteolytically cleaved to generate p25, and the resultant CDK5/p25 complex is critically involved in neuronal development, differentiation, and synaptic remodeling regulation. Our findings indicate that SHPL-49 enhances synaptic remodeling and exerts neuroprotective effects through the upregulation of both CDK5 mRNA and protein expression levels.

### 3.5 SHPL-49 promotes synaptic plasticity by inhibiting the proteolytic cleavage of p35 into p25

**FIGURE 5:**
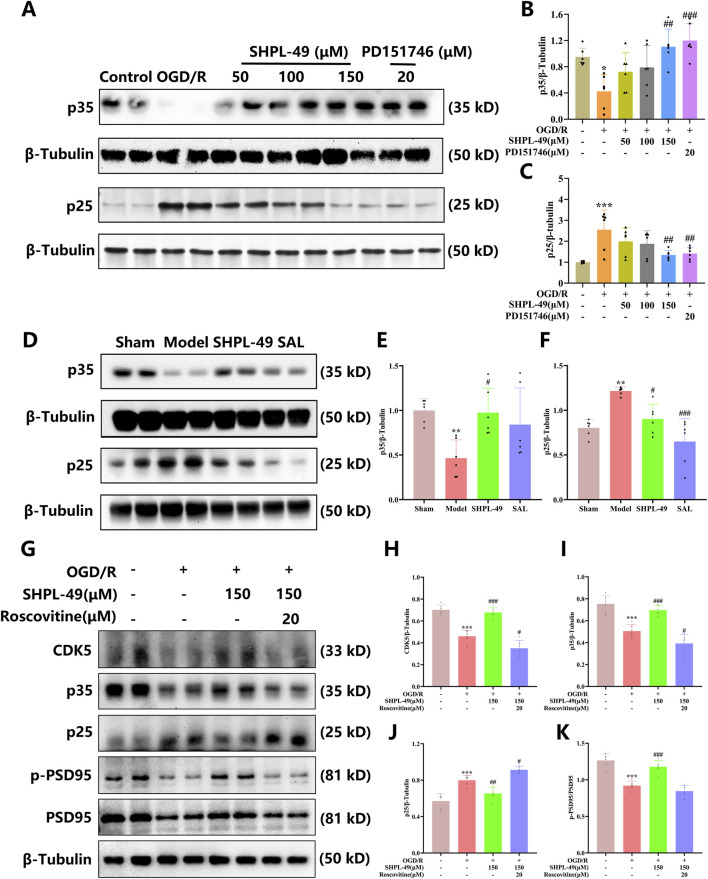
SHPL-49 inhibits the proteolytic conversion of p35 to p25. **(A)** Representative Western blot images showing p35 and p25 expression in primary neurons. **(B)** Quantitative analysis of p35 protein lev-els in primary neurons. **(C)** Quantitative analysis of p25 protein levels in primary neurons. **(D)** Representative Western blot images of p35 and p25 in rat brain tissue. **(E)** Quantitative analysis of p35 protein levels in rat brain tissue. **(F)** Quantitative analysis of p25 protein levels in rat brain tis-sue. **(G)** Representative Western blot images showing the expression levels of CDK5, p35, p25, and p-PSD95/PSD95 ratio in primary neurons following OGD/R treatment and co-treatment with SHPL-49 and CDK5 inhibitor Roscovitine. **(H)** Quantitative analysis of CDK5 protein levels in primary neurons under the same experimental conditions. **(I)** Quantitative analysis of p35 protein levels in primary neurons under the same experimental conditions. **(J)** Quantitative analysis of p25 protein levels in primary neurons under the same experimental conditions. **(K)** Quantitative analysis of p-PSD95/PSD95 ratio in primary neurons under the same experimental conditions. Data are presented as mean ± SD. *P < 0.05, **P < 0.01, ***P < 0.001, OGD/R group vs. Control group, Model group vs. Sham group, ^#^ *P* < 0.05, ^##^ *P* < 0.01, ^###^ *P* < 0.001, SHPL-49 group or PD151746 group vs. OGD/R group, SHPL-49 group or SAL group vs. Model group n = 6 per group.

CDK5r1 regulates the production of p35, a specific activator of CDK5. Under cerebral ischemia conditions, p35 undergoes proteolytic cleavage to form p25 (Maitra and Vincent, 2022). To investigate SHPL-49 modulates the expression levels of p35 and p25 proteins, we performed a Western blot analysis. Under OGD/R conditions, after treating primary neurons with SHPL-49 at concentrations of 50 μM, 100 μM, and 150 μM, the protein expression level of p35 upregulated, while that of p25 decreased correspondingly. Notably, treatment with 150 μM SHPL-49 resulted in statistically significant effects (P < 0.01). The positive control PD151746 (20 μM) effectively prevented the cleavage of p35 into p25, and 150 μM SHPL-49 produced a comparable inhibitory effect (Figures 5A–C). These findings suggest that SHPL-49 attenuates OGD/R-induced p35 proteolysis in primary cortical neurons, thereby preserving full-length p35 and reducing accumulation of the neurotoxic p25 fragment. Furthermore, in brain tissue obtained from BCCAO rats, SHPL-49 treatment significantly increased p35 protein expression (*P* < 0.05), whereas the increase in p35 expression observed in the SAL group was not statistically significant. The protein expression of p25 was significantly reduced in the SHPL-49 group (*P* < 0.05, *P* < 0.001) (Figures 5D–F). These findings indicate that SHPL-49 markedly enhances the expression of p35 protein while significantly suppressing the production of p25 protein in the brain tissue of BCCAO rats. This effect aligns with the findings observed in primary neurons. In summary, SHPL-49 promotes the expression of p35, a specific activator of CDK5, and inhibits its proteolytic cleavage into p25, thereby preventing the aberrant activation of CDK5. This regulatory mechanism facilitates synaptic remodeling in neurons and ultimately confers neuroprotective benefits.

To investigate whether SHPL-49 exerts its effects via the CDK5 pathway, we examined expression changes of key proteins in primary neurons using the OGD/R model through Western blotting. The expression levels of CDK5, p35, and p25 proteins were consistent with previous findings. Additionally, SHPL-49 increased PSD95 expression and simultaneously raised the phosphorylation level of PSD95, a downstream target of CDK5 (*P* < 0.001). However, when the CDK5 inhibitor Roscovitine (20 μM) was co-administered, the regulatory effects of SHPL-49 on these proteins were blocked or reversed. In the presence of the CDK5 inhibitor Roscovitine, SHPL-49 failed to significantly alter the protein levels of CDK5, p35, and p25, and the SHPL-49 induced increase in the p-PSD95/PSD95 ratio was completely abolished (Figures 5G–K). These results demonstrate that SHPL-49 exerts its effects on postsynaptic protein phosphorylation and expression following OGD/R injury specifically through modulation of the CDK5/p35/p25 signaling axis - supporting synaptic remodeling and contributing to its neuroprotective effects.

To further investigate the mechanism underlying SHPL-49 SHPL-49 - mediated inhibition of p35 cleavage, the calpain enzymatic activity was quantified in both the brain tissue of BCCAO rats and in primary neurons subjected to OGD/R. As shown in Supplementary Figure S1A, calpain activity was significantly elevated in the BCCAO group relative to the sham-operated control group (*P* < 0.001). Administration of SHPL-49 (90 mg/kg) significantly reduced the calpain activity (*P* < 0.001). Consistent with the *in vivo* findings, calpain enzymatic activity was significantly elevated in the primary neurons following OGD/R (*P* < 0.001), and SHPL-49 treatment significantly attenuated this increase (*P* < 0.001). Notably, SHPL-49 at 150 μM achieved inhibition comparable to that of the selective calpain inhibitor PD151746 (20 μM) (Supplementary Figure S1B). These results indicate that SHPL-49 inhibits calpain-mediated protein hydrolysis, thereby preserving p35 integrity and preventing its pathological cleavage into the neurotoxic p25 fragment.

## 4 Discussion

Long-term neurological deficits following ischemic stroke represent a major clinical challenge in the field of neural repair, commonly manifesting as persistent cognitive impairments and motor coordination dysfunction (Kohara et al., 2025). Cognitive deficits, particularly those involving declarative memory loss, are primarily attributed to the disruption of hippocampal structural integrity, which compromises the processes of memory encoding and consolidation (Rost et al., 2022). Motor dysfunction is characterized by hemiplegia, diminished fine motor control, and gait disturbances, frequently associated with impaired hippocampus-dependent spatial navigation and damage to the motor cortex (Wood et al., 2024). Notably, synaptic remodeling plays a pivotal role in neurological recovery following stroke (Beker et al., 2025). In the cognitive domain, hippocampal synaptic remodeling is essential for the restoration of learning and memory functions (Wang et al., 2023). With regard to motor recovery, promoting synaptic remodeling in both the hippocampus and the motor cortex, as well as strengthening neuronal connectivity, contributes to improved limb coordination (Chen et al., 2023). This reparative process is histologically evident through axonal sprouting, increased dendritic arborization, and the formation of new dendritic spines (Xing and Bai, 2020).

Previous studies have demonstrated that SHPL-49 significantly improves cognitive and motor functions in the pMCAO model, exhibiting robust neuroprotective effects (Zhang et al., 2023). The pMCAO model primarily induces acute focal ischemia, leading to cortical infarction and resulting in cognitive and motor deficits (Pereira et al., 2023). In contrast, the BCCAO model more accurately recapitulates the progressive hippocampal damage associated with chronic cerebral hypoperfusion, thereby modeling a distinct post-stroke pathological process characterized by long-term functional decline (Ma et al., 2021). Therefore, further investigation into the effects of SHPL-49 in the BCCAO model is crucial for evaluating its long-term therapeutic potential. Such studies may determine whether SHPL-49 confers protection against neurodegenerative changes induced by sustained hypoperfusion, thereby providing more comprehensive preclinical evidence to support its development as a multi-target neuroprotective agent.

Therefore, in this study, using a well-established BCCAO rat model, we evaluated for the first time the neuroprotective effects of long-term intragastric administration of SHPL-49 on neurological deficits. Following 28 days of continuous administration, SHPL-49 significantly improved long-term memory between day 24 and day 28, as assessed by the Morris water maze. Specifically, the latency to locate the hidden platform was significantly reduced, the time spent in the target quadrant during the probe trial was significantly increased, and the distance traveled to reach the target quadrant was significantly decreased in the SHPL-49 group compared to the model group. Furthermore, in the shuttle box experiment, the SHPL-49 group exhibited significantly fewer errors on days 21 and 28 compared to the model group. These findings suggest that SHPL-49 enhances learning acquisition and memory retention following ischemic stroke. Additionally, motor function was evaluated using running wheel experiments, the rota-rod test, and gait analysis. Notably, the collective results indicate that SHPL-49 ameliorates motor deficits, including impairments in flexibility, balance, and coordination, in rats subjected to ischemic stroke. Although the present study comprehensively evaluated the effects of SHPL-49 on cognitive and motor recovery following ischemic stroke using a battery of validated behavioral assays, additional paradigms, including novel object recognition, novel location recognition, and contextual fear conditioning - could further delineate its impact on distinct cognitive domains such as episodic memory, spatial memory, and associative learning. These complementary approaches are planned for future investigation to strengthen mechanistic interpretation and translational relevance.

The hippocampus plays a critical role in learning and memory through the activity of neural circuit (Veschsanit et al., 2021). Neuronal damage in the hippocampal CA3 region is a prominent pathological feature following ischemic stroke and may contribute to cognitive and motor impairments (Montoya-García et al., 2023). Our findings demonstrate that long-term administration of SHPL-49 significantly reduces ischemia-induced neuronal damage in the hippocampal CA3 region, as evidenced by H&E and Nissl straining and NeuN immunofluorescence staining. Behaviorally, the improvement of learning and motor coordination relies on enhanced synaptic transmission efficiency and functional reorganization of neural networks. The result of Golgi staining demonstrates that SHPL-49 increases dendritic spine density, thereby promoting neuronal morphological integrity. SYP is a synaptic vesicle membrane-integrated glycoprotein, and SYN1 is a glycoprotein localized to the synaptic vesicle membrane. Both serve as the core marker molecules of the presynaptic terminal. PSD95 is a central scaffold protein within the postsynaptic density region and a key molecule marker of the postsynaptic membrane (Cannata et al., 2025; Sun et al., 2025). These proteins are essential indicators for assessing presynaptic functional integrity and postsynaptic membrane structural preservation. As expected, SHPL-49 can increase the protein expression of SYP, SYN1 and PSD95 after cerebral ischemia. Gliotic scars impede synaptic reconstruction and functional recovery by releasing inhibitory molecules, such as chondroitin sulfate proteoglycans (CSPGs). SHPL-49 mitigates gliotic scar formation and enhances synaptic remodeling following cerebral ischemia. To the best of our knowledge, this study is the first to emphasize that SHPL-49 plays a key role in mediating synaptic remodeling. However, this study still has certain limitations. In investigating the effects of SHPL-49 on synaptic structure and function, we employed classical methodologies, including Golgi-Cox staining and transmission electron microscopy (TEM). Although Golgi-Cox staining enables direct quantification of dendritic spine number, density, and morphological diversity, and TEM allows visualization of synaptic ultrastructure, the latter is limited by a narrow field of view, which restricts comprehensive observation of neural network architecture (Wang et al., 2025c). Given our primary focus on SHPL-49-mediated promotion of synaptic growth, Golgi-Cox staining was selected as the principal methodological approach. Nevertheless, integration of TEM data would enhance the comprehensive of the analysis. In future studies, we aim to further investigate the underlying mechanisms governing synaptic structure and function.

In the central nervous system, cyclin-dependent kinase 5 (CDK5), a member of the cyclin-dependent kinase (CDK) family, is predominantly expressed in neurons and plays a critical regulatory role in neuronal survival and apoptosis (Ao et al., 2022; Requejo-Aguilar, 2023). It modulates synaptic transmission by phosphorylating key substrates at both presynaptic and postsynaptic sites, a process often coordinated with the activity of the phosphatase calcineurin (Wang et al., 2020; Wang et al., 2025a). The active complex formed by CDK5 and its activator p35 is essential for fundamental neuronal processes, including neuronal survival, synaptic development and function, memory consolidation, and motor performance enhancement (Jahan et al., 2023; Jenkins and Bender, 2025). Under pathological conditions, p35 can be proteolytically cleaved to generate p25, resulting in sustained activation of CDK5 and potential contribution to neurodegenerative processes (Kao et al., 2025; Tran et al., 2021). The resulting CDK5/p25 complex is highly stable and implicated in various pathological processes, including synaptic dysfunction and neuronal apoptosis (Rabie et al., 2025; Simhadri et al., 2025). This study investigated the neuroprotective effects of SHPL-49 in a rat model of BCCAO and demonstrated that SHPL-49 significantly promoted synaptic remodeling by inhibiting the CDK5/p25 signaling pathway. Moreover, SHPL-49 further enhanced synaptic remodeling following cerebral ischemia by upregulating the expression of p35, a specific activator of CDK5, and suppressing its proteolytic cleavage to p25. These effects ultimately contributed to improved cognitive and motor functions in ischemic stroke, indicating the neuroprotective potential of SHPL-49. To further delineate the essential role of CDK5 in SHPL-49 mediated enhancement of synaptic plasticity, we pharmacologically inhibited CDK5 using CDK5-specific inhibitor Roscovitine. Consistent with our hypothesis, Roscovitine pretreatment not only abolished the SHPL-49 induced upregulation of CDK5 protein levels but also markedly attenuated its regulatory effects on p35 and p25 expression, as well as on the phosphorylation status of the downstream synaptic scaffold protein PSD95 (p-PSD95/PSD95 ratio). Collectively, these findings indicate that SHPL-49 promotes synaptic remodeling through a dual mechanism: by sustaining CDK5 protein expression and by modulating its kinase activity - both of which are indispensable for its functional impact on postsynaptic signaling. Collectively, these findings confirm that SHPL-49 confers neuroprotection through modulation of the CDK5/p35/p25 signaling axis. While direct quantification of CDK5 kinase activity would further substantiate this mechanism, the convergence of multiple orthogonal lines of evidence, including phosphatase substrate profiling (e.g., p-PSD95), pharmacological rescue with Roscovitine, and calpain activity assays - robustly supports the conclusion that SHPL-49 promotes synaptic resilience through coordinated regulation of this pathway. Transcriptomic analysis identified synaptic remodeling - associated pathways as top enriched signatures; however, upstream transcriptional regulators, including potential transcription factors and epigenetic modulators remain to be characterized. The future will delve deeper into the neuroprotective effects of SHPL-49. However, this study has certain limitations. Although PC-12 cells exhibit neuron-like properties, they lack authentic synaptic structures and therefore cannot fully recapitulate the physiological and functional complexity of primary neurons or accurately reflect *in vivo* synaptic remodeling. Compared to primary embryonic spinal cord cultures, PC-12 cells display significantly restricted transcriptional responses following NGF stimulation (Pazman et al., 2000). This highlights the inherent limitations of cell line models in mimicking the transcriptional regulation associated with pathophysiological conditions. Our future research will concentrate on performing more systematic multi-omics analyses using primary neuronal cultures and BCCAO animal models, which more closely resemble the *in vivo* pathophysiological environment. This study aims to comprehensively and precisely elucidate the molecular mechanism underlying SHPL-49-mediated synaptic repair and to evaluate its therapeutic potential for neural function recovery following ischemic stroke.

**FIGURE 6:**
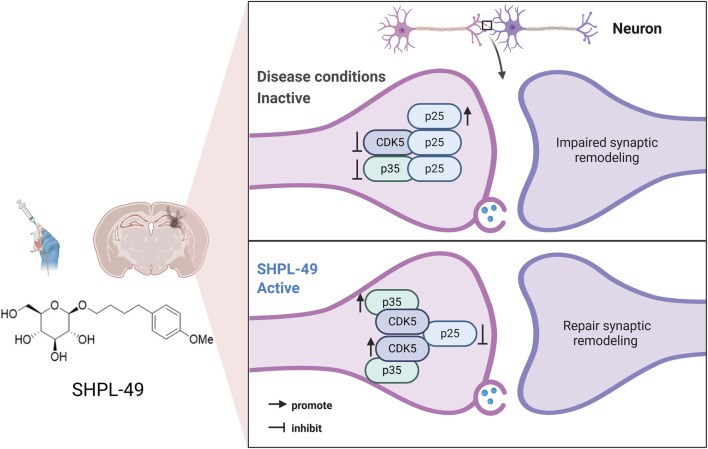
Schematic illustration of the neuroprotective effect of SHPL-49 in ischemic stroke by promoting synaptic remodeling.

In summary, our findings demonstrate that SHPL-49 alleviates cognitive and motor impairments following ischemic stroke by promoting synaptic remodeling (Figure 6). Furthermore, SHPL-49 exerts neuroprotective effects through modulation of the CDK5/p35/p25 signaling pathway, thereby enhancing synaptic remodeling in neuronal cells. Although we have identified that synaptic remodeling in animal models of ischemic stroke following long-term administration of SHPL-49 is regulated through the CDK5/p35/p25 signaling pathway, the underlying molecular mechanisms remain to be fully elucidated. Multiple signaling pathways have been demonstrated to play critical roles in synaptic remodeling within the nervous system. The Wnt/β-catenin pathway is centrally involved in the regulation of synaptic morphology and structural reorganizatio (Lv et al., 2023); the Notch pathway significantly contributes to synaptic maintenance and adaptive remodeling (Wu et al., 2021); furthermore, the mTOR pathway is essential for synaptic growth and plasticity associated with learning and memory (Yuan et al., 2022). Therefore, to comprehensively elucidate the mechanism of SHPL-49 in synaptic remodeling, precisely identify its molecular targets, and establish a robust pharmacological and safety foundation for clinical translation, more in-depth research should be conducted across a broader range of experimental models. This remains a critical direction for future investigation.

## Data Availability

The full western blot membranes are provided in Supplementary Data Sheet 1. The sequencing data generated in this study have been deposited in the Genome Sequence Archive (GSA) repository under accession number CRA039936.
