# Supplementary material for: Salidroside derivative SHPL-49 enhances synaptic remodeling in BCCAO rats via the CDK5/p35/p25 signaling pathway
Source: Front Pharmacol. 2026 Mar 23;17:1727177. doi: 10.3389/fphar.2026.1727177 (PMC13050835; doi:10.3389/fphar.2026.1727177)
Supplement: Supplementary file 1 [file Supplementaryfile1.docx]

Fig. S1


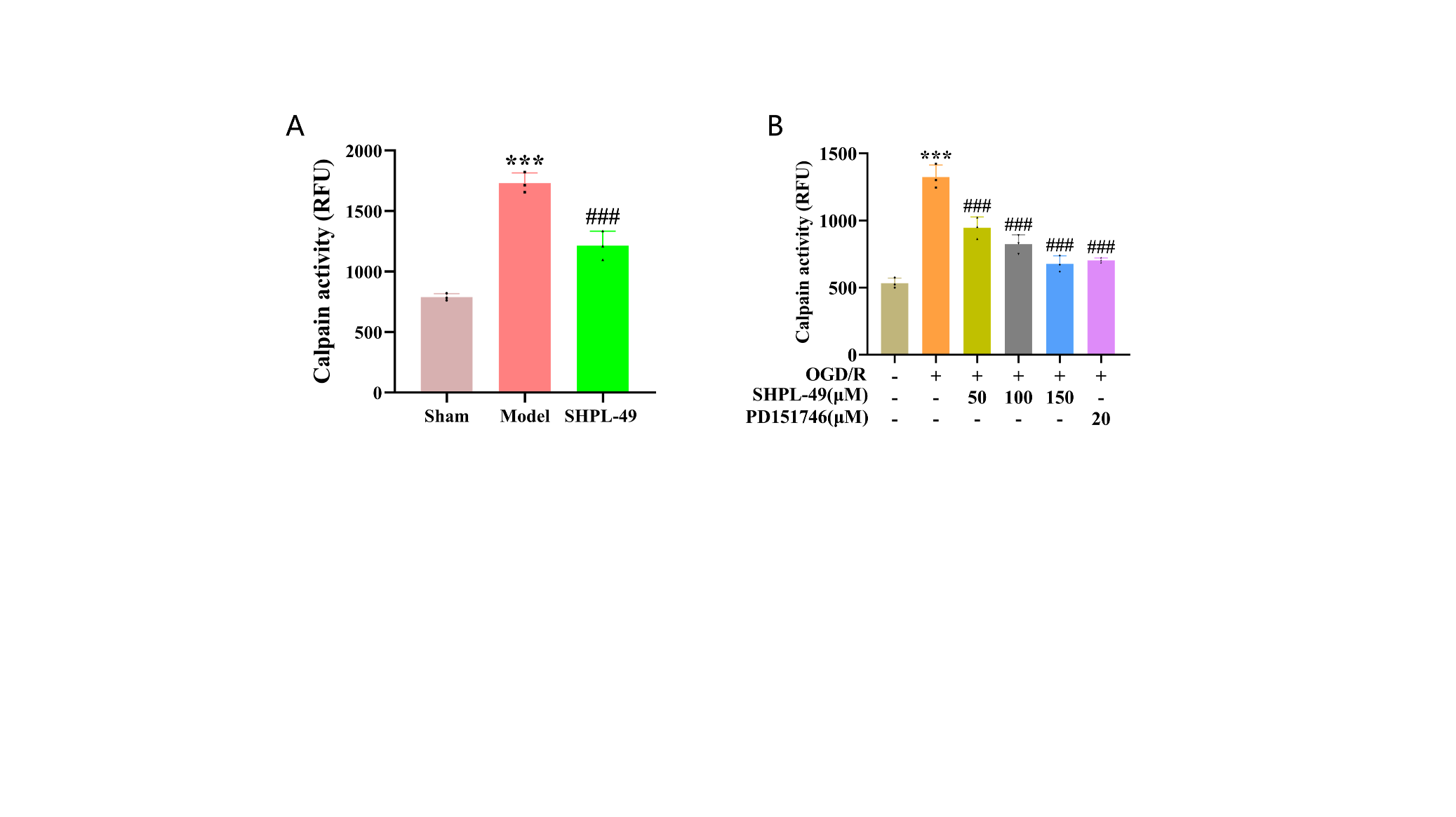


SHPL-49 inhibits the Calpain activity in BCCAO rats and primary neurons after OGD/R treatment (A) Calpain activity in BCCAO rat brain tissues (B) Calpain activity in primary neurons after OGD/R treatment. Data are expressed as mean ± SD. *** *P* < 0.001, Model group vs. Sham group; ### *P* < 0.001, SHPL-49 group or SAL group vs. Model grou. n = 3 per group.

Fig. S2


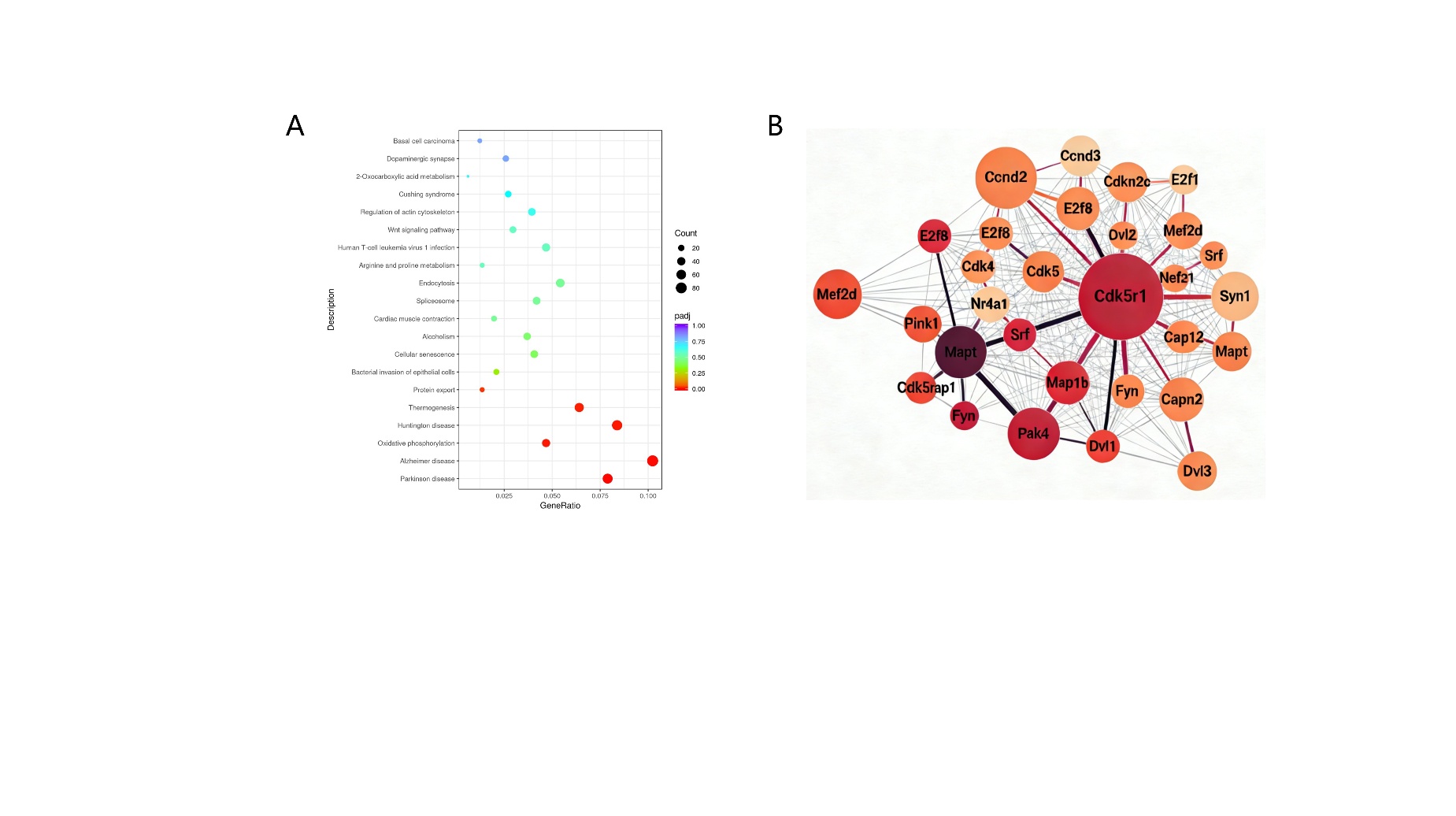


SHPL-49 promotes the expression of CDK5 at both mRNA and protein levels. (A) KEGG enrichment analysis of differentially expressed genes (B) PPI protein network diagram. Control group, n = 4; SHPL-49 group, n = 5.
