# Supplementary material for: Salidroside derivative SHPL-49 enhances synaptic remodeling in BCCAO rats via the CDK5/p35/p25 signaling pathway
Source: Front Pharmacol. 2026 Mar 23;17:1727177. doi: 10.3389/fphar.2026.1727177 (PMC13050835; doi:10.3389/fphar.2026.1727177)

Fig. 3F

Western blot images showing SYP expression levels in rat brain tissue (n=6)

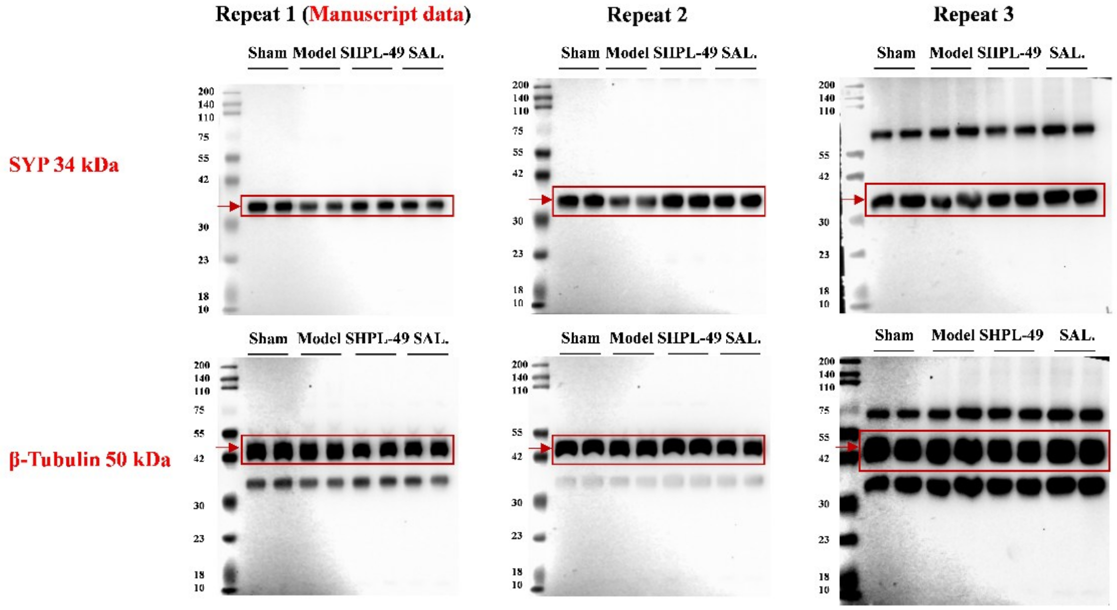

Fig. 3F

Western blot images showing SYN1 expression levels in rat brain tissue (n=6)

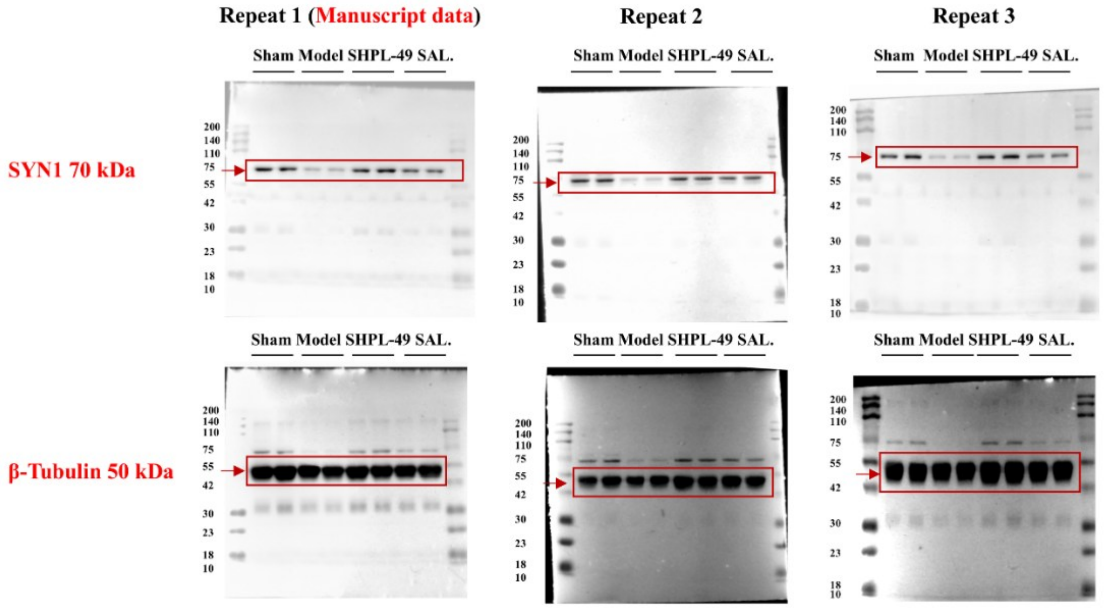

Fig. 3F

Western blot images showing PSD95 expression levels in rat brain tissue (n=6)

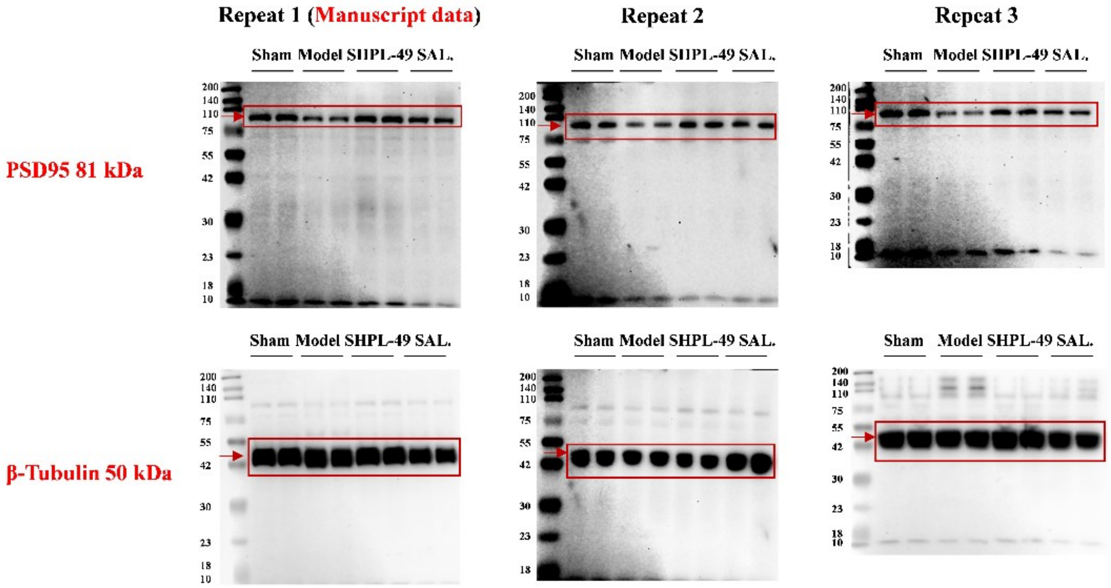

Fig. 4O

Western blot images showing of CDK5 expression levels in primary neurons (n=6)

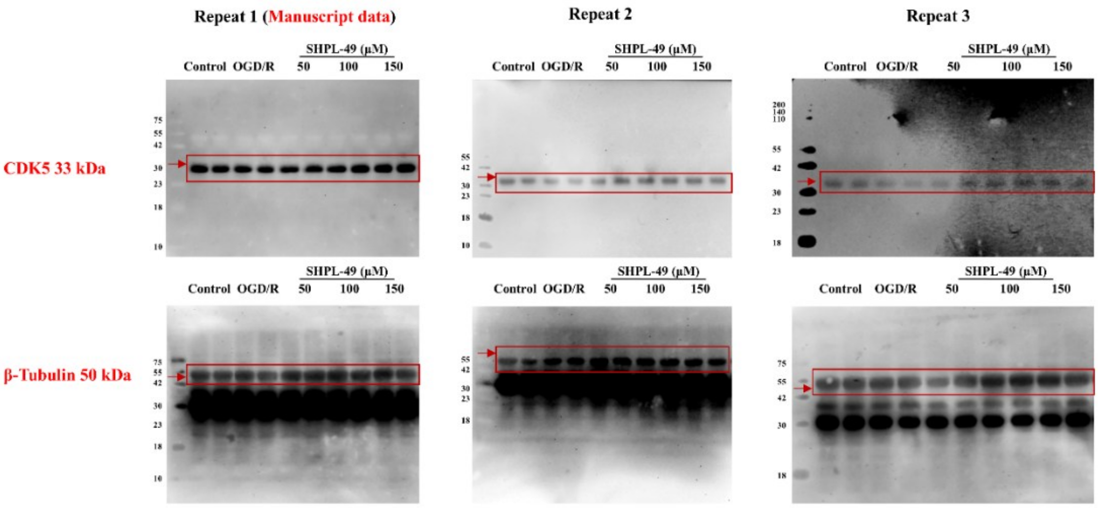

Fig. 4Q

Western blot images showing CDK5 expression levels in rat brain tissue (n=6)

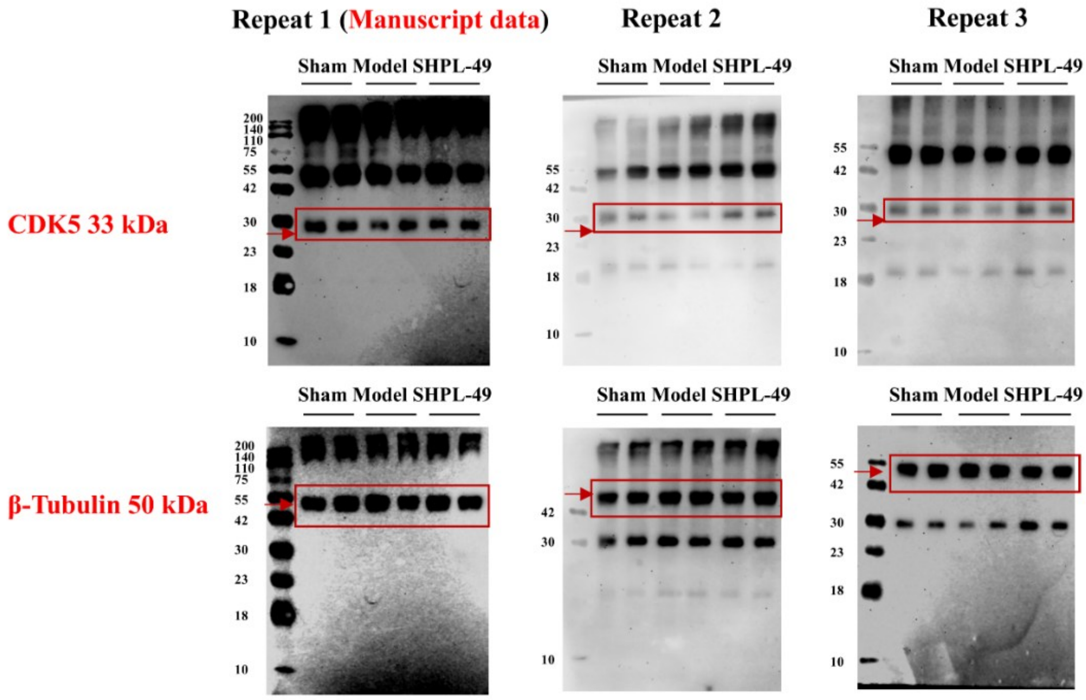

Fig. 5A

Western blot images showing p35 expression levels in primary neurons (n=6)

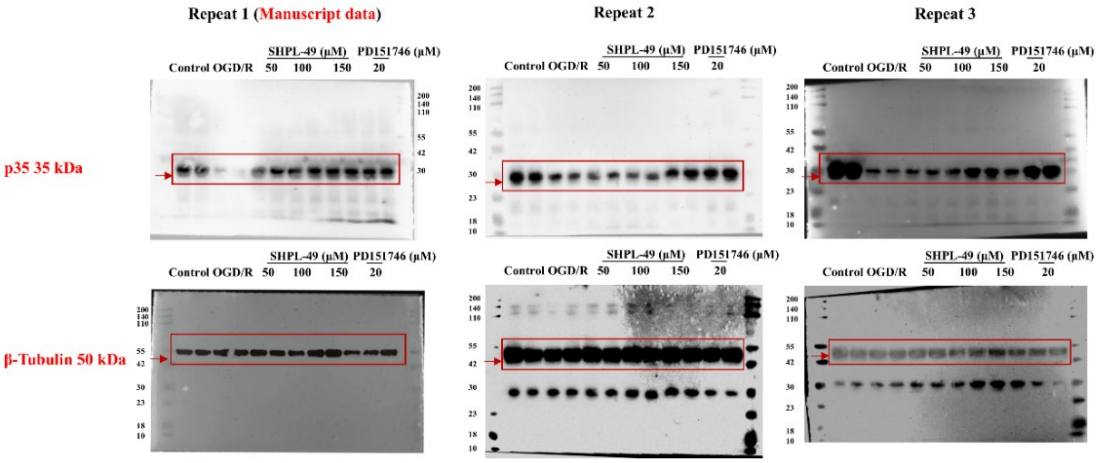

Fig. 5A

Western blot images showing p25 expression levels in primary neurons (n=6)

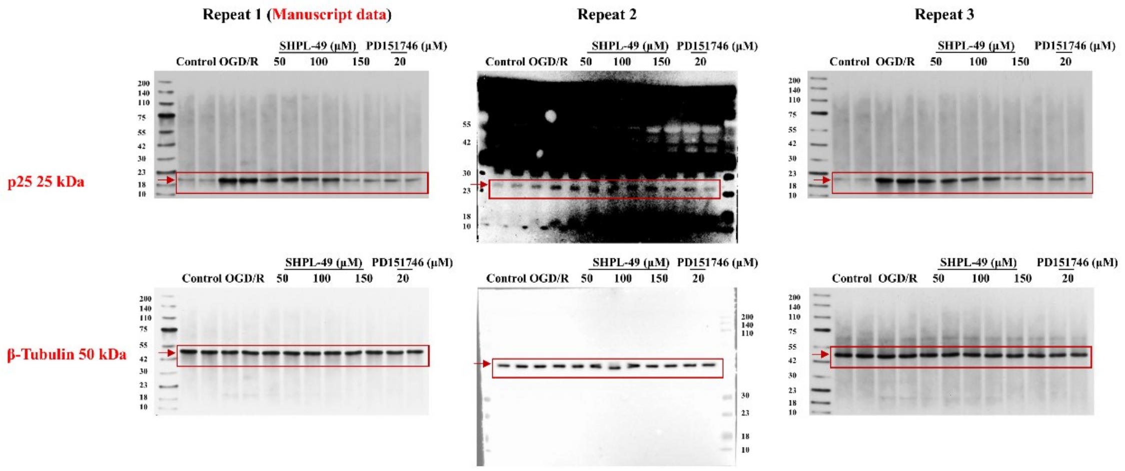

Fig. 5D

Western blot images showing p35 expression levels in rat brain tissue (n=6)

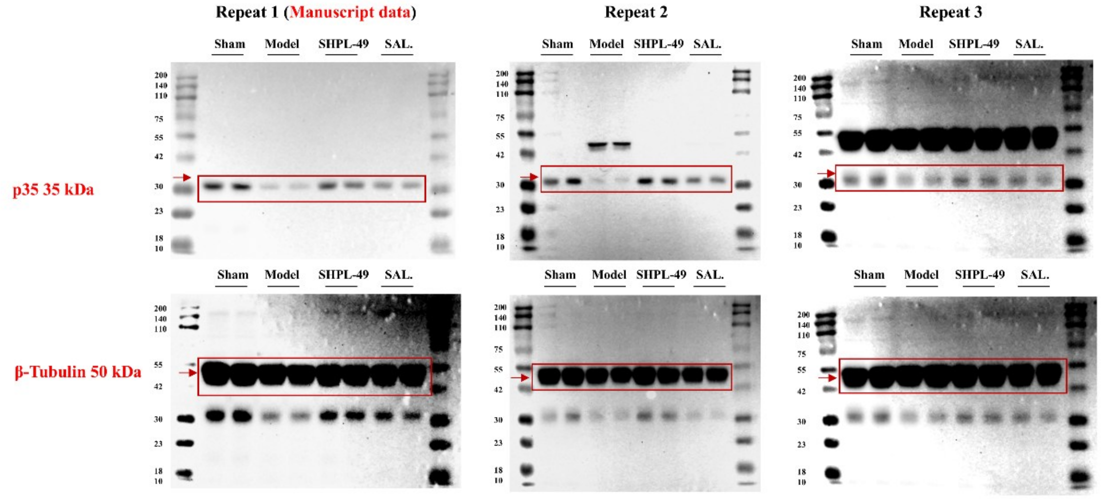

Fig. 5D

Western blot images showing p25 expression levels in rat brain tissue (n=6)

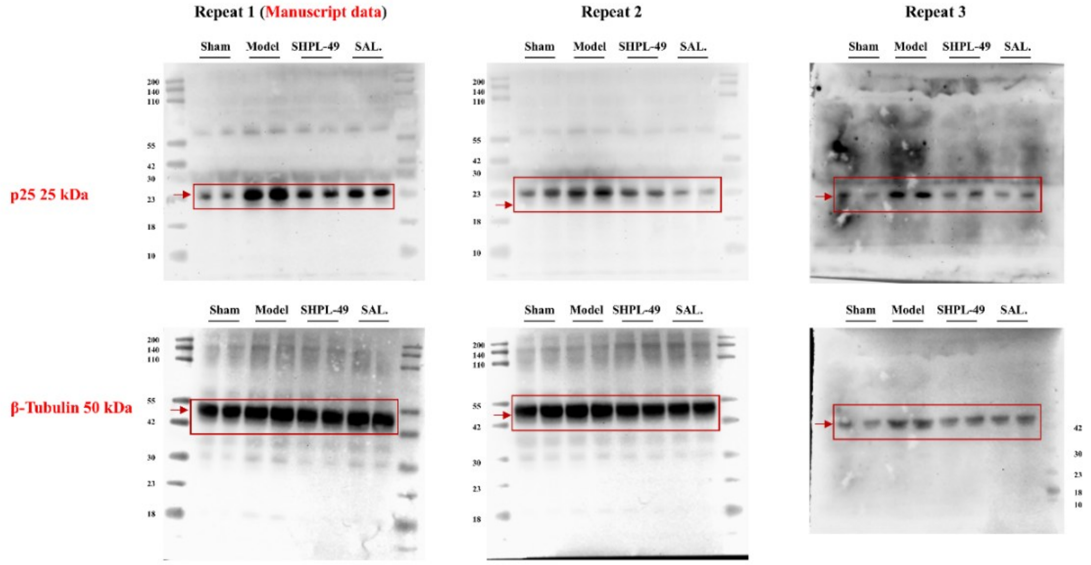

Fig. 5G

Western blot images showing CDK5 expression levels in primary neurons (n=6)

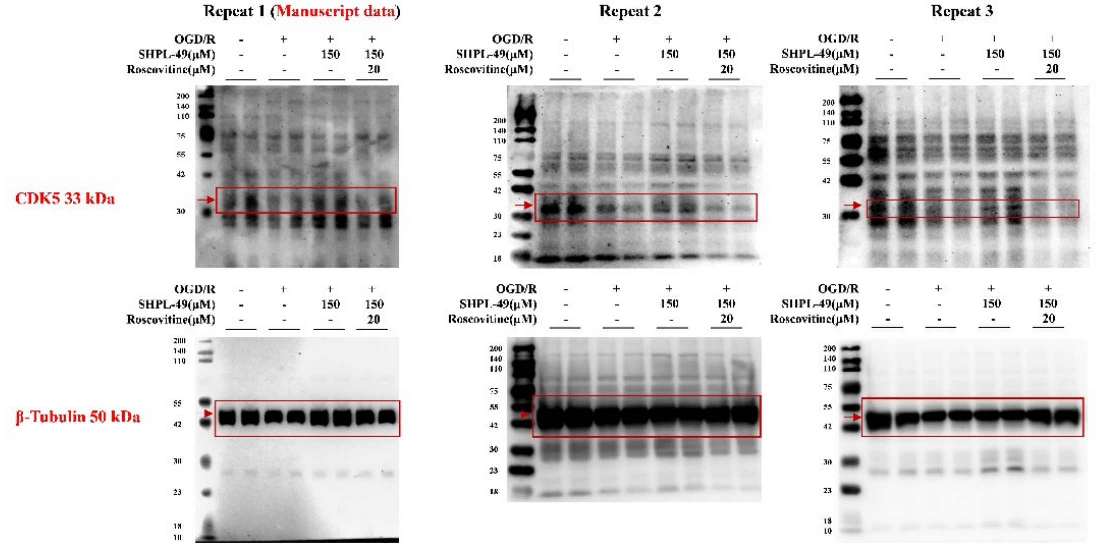

Fig. 5G

Western blot images showing p35 expression levels in primary neurons (n=6)

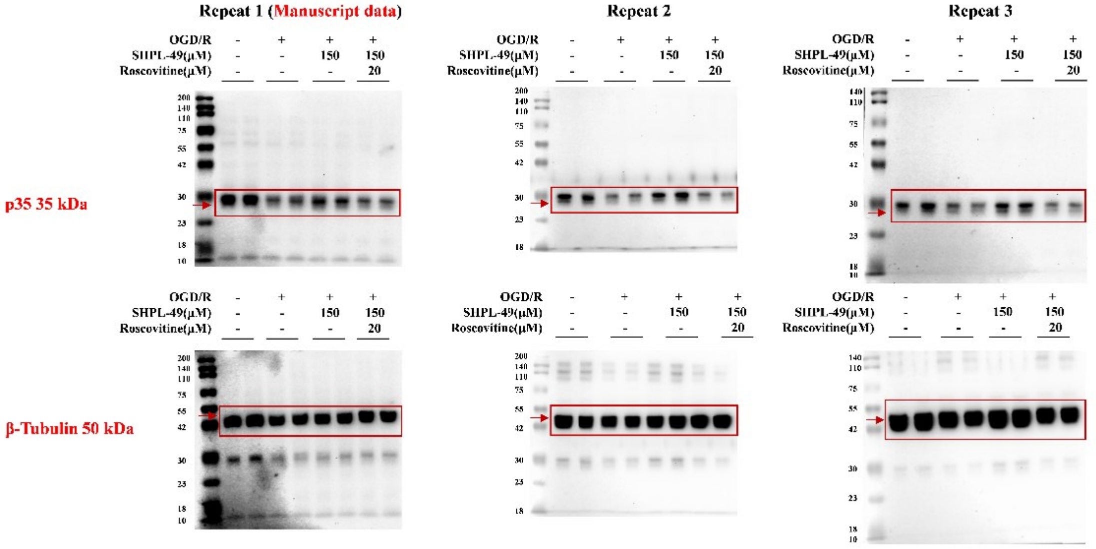

Fig. 5G

Western blot images showing p25 expression levels in primary neurons (n=6)

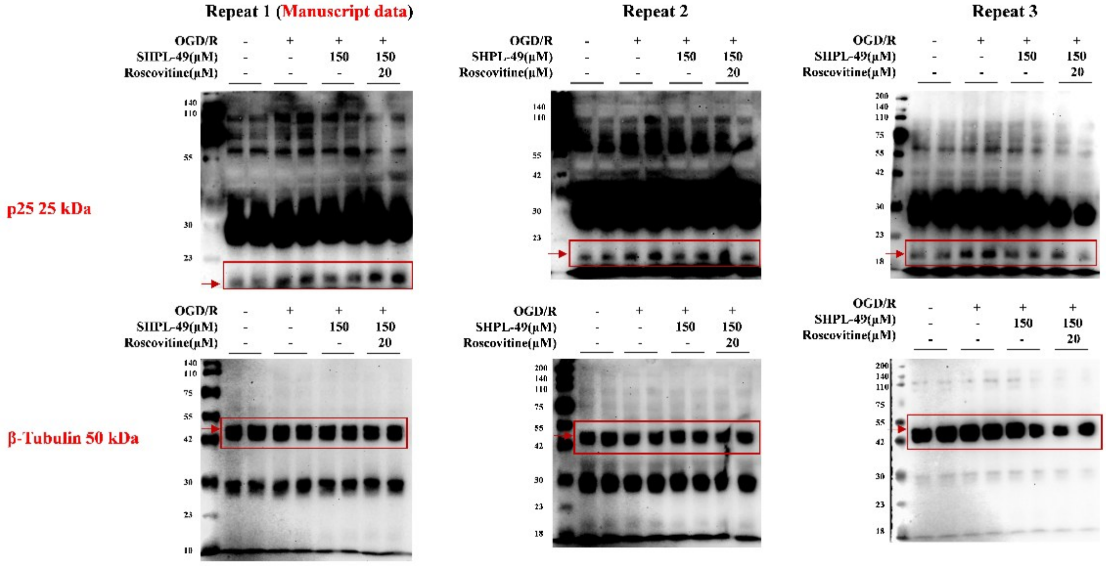

Fig. 5G

Western blot images showing p-PSD95 expression levels in primary neurons (n=6)

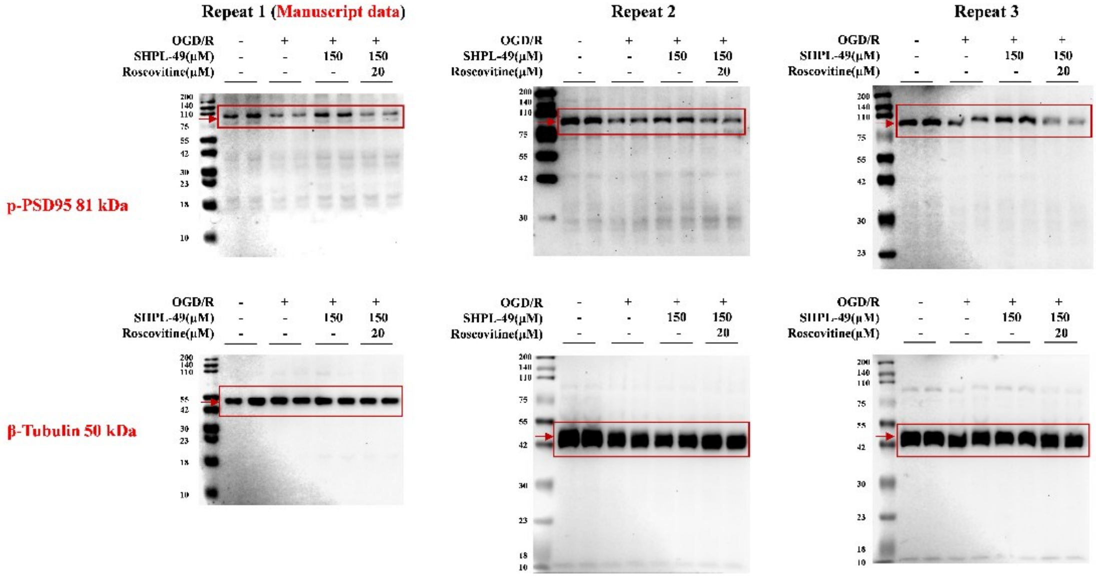

Fig. 5G

Western blot images showing PSD95 expression levels in primary neurons (n=6)

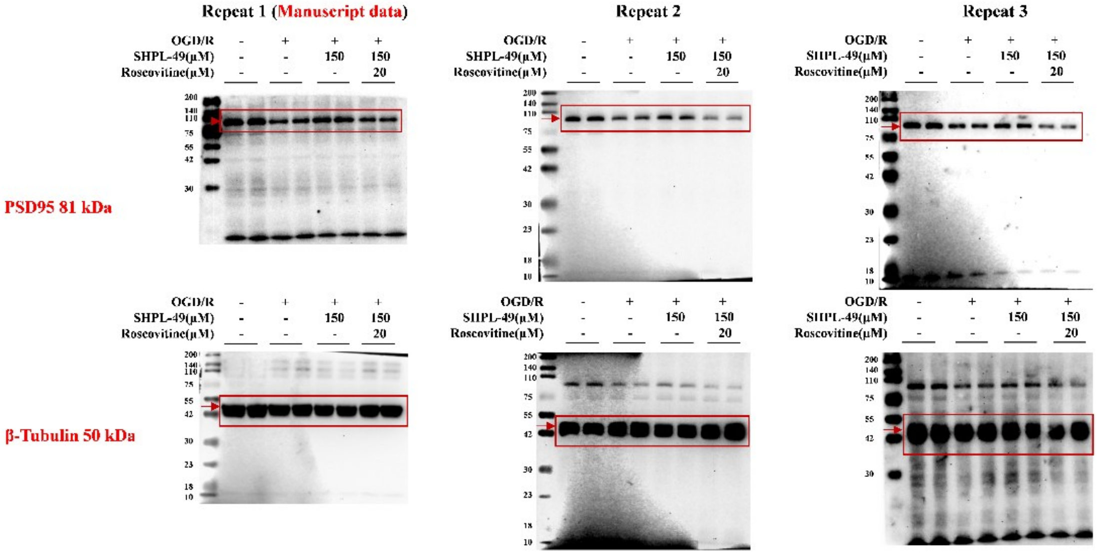

Supplement: Supplementary file 2 [file DataSheet1.pdf]
